# System, Design and Experimental Validation of Autonomous Vehicle in an Unconstrained Environment

**DOI:** 10.3390/s20215999

**Published:** 2020-10-22

**Authors:** Shoaib Azam, Farzeen Munir, Ahmad Muqeem Sheri, Joonmo Kim, Moongu Jeon

**Affiliations:** 1 School of Electrical Engineering and Computer Science, Gwangju Institute of Science and Technology (GIST), Gwangju 61005, Korea; shoaibazam@gist.ac.kr (S.A.); farzeen.munir@gist.ac.kr (F.M.); 2 Department of Computer Software Engineering, National University of Sciences and Technology (NUST), Islamabad 44000, Pakistan; muqeem@mcs.edu.pk; 3 Department of Computer Engineering, Dankook University, Gyeonggi-do 16890, Korea; q888@dankook.ac.kr

**Keywords:** autonomous vehicle system, intelligent transportation system, autonomous taxi service, localization, perception, mission and motion planning, control

## Abstract

In recent years, technological advancements have made a promising impact on the development of autonomous vehicles. The evolution of electric vehicles, development of state-of-the-art sensors, and advances in artificial intelligence have provided necessary tools for the academia and industry to develop the prototypes of autonomous vehicles that enhance the road safety and traffic efficiency. The increase in the deployment of sensors for the autonomous vehicle, make it less cost-effective to be utilized by the consumer. This work focuses on the development of full-stack autonomous vehicle using the limited amount of sensors suite. The architecture aspect of the autonomous vehicle is categorized into four layers that include sensor layer, perception layer, planning layer and control layer. In the sensor layer, the integration of exteroceptive and proprioceptive sensors on the autonomous vehicle are presented. The perception of the environment in term localization and detection using exteroceptive sensors are included in the perception layer. In the planning layer, algorithms for mission and motion planning are illustrated by incorporating the route information, velocity replanning and obstacle avoidance. The control layer constitutes lateral and longitudinal control for the autonomous vehicle. For the verification of the proposed system, the autonomous vehicle is tested in an unconstrained environment. The experimentation results show the efficacy of each module, including localization, object detection, mission and motion planning, obstacle avoidance, velocity replanning, lateral and longitudinal control. Further, in order to demonstrate the experimental validation and the application aspect of the autonomous vehicle, the proposed system is tested as an autonomous taxi service.

## 1. Introduction

The development of autonomous vehicles has seen a tremendous interest both from academia and industry. This significant progress in the autonomous vehicles has benefits in terms of mobilizing traffic capacity, vehicular emission and safety as a surrogate to conventional vehicles. Although the advance of autonomous vehicle is unassailable, the safety of the autonomous systems is of prime concern. The deployment of the autonomous vehicle in an urban environment is challenging and must accomplish the safety measures as standardized by SOTIF-ISO/PAS-21448 (Safety of the intended functionality). However, the advancements in the vehicle dynamics, incorporation of deep learning for vision tasks and affordability of new sensor modalities have catalyzed the autonomous driving research and safety protocols.

The concept of the autonomous vehicle has revolutionized for 100 years. In 1920s, “Phantom Auto” is articulated, which is the earliest attempt of autonomous vehicle [1]. A demonstration is performed by Achen motor company, in which the car is driven without a driver, by controlling through radio communication. Since then, the autonomous vehicle has evolved from using street marker tracing to control the car to cruise control [2], and autopilot [3]. Many stakeholders and academic research groups are striving to compete with the growing domain of the autonomous vehicle. Despite this towering advancement and progress, autonomous vehicles lack human-level intelligence, and cognition [4], and to achieve that goal, many research groups have developed their autonomous vehicles test-beds [5,6,7,8,9,10,11]. The most influential companies perusing autonomous vehicle dream include Uber [12], Tesla [13], Waymo [14], GM [15], and Apollo [10].

Besides, the efforts have started in 1920s for the autonomous vehicle, the impact of DARPA autonomous driving competition has dramatically revolutionized the field of autonomous vehicles. The whole challenge is a big step towards autonomous driving, and the illustrious vehicles that are produced as an outcome of this competition are briefly discussed below. In 2005 the first DARPA competition winner, Stanley is created by the Stanford Racing Team in collaboration with Volkswagen Electronics Research Laboratory (ERL) [16]. Stanley is a four-wheel diesel-powered Volkswagen Touareg R5 with variable height suspension and automatic electronic locking differential, and it was arrayed with five SICK laser range finder, color camera and two 24 GHz radars for environmental perception. Its navigation module comprises an inertial measurement unit (IMU), and Global Positioning System (GPS). The software architecture of Stanley is composed of six modules: sensors interface, perception, control, user interface, vehicle interface, and global services. The 2007 DARPA Urban Challenge winner, Boss is presented by Carnegie Mellon University [17]. The sensory network of Boss is equipped with the global positioning system, Lidars, radars, and cameras for localization, the perception of the environment and motion planning, respectively. The whole architecture is divided into three main modules: mission, motion, and behavioural planning. Similarly, other research labs have also made efforts to develop their autonomous car test-bed. The Karlsruhe Institute of Technology collaborating with Toyota Technological Institute Chicago launched a project named AnnieWAY, which is equipped with four cameras, a 3D laser scanner, and a Global Positioning System (GPS), and Inertial Measurement Unit (IMU) navigation system. The prospective advantage of their autonomous platform is in the form of the novel KITTI dataset that is utilize for mobile robotics and autonomous driving research [18,19]. RobotCar from the University of Oxford is an autonomous Nissan LEAF equipped with six cameras along with Lidar and GNSS (Global Navigation and Satellite System) system [20]. Using their autonomous platform, they have published a challenging dataset for autonomous driving comprising all of the weather conditions. Other notable autonomous cars are MIT Human-Centered Autonomous Car [21], Modified Ford F-250 pickup truck [22] and Spirit of Berlin [23].

The Society of Automobile Engineers (SAE) has specified six levels of automation, with level 0 corresponds to no automation features present in the vehicle to level 5 complete automation. The increase in automation level demands the deployment of robust sensor suite and algorithms. The three main subsystems that constitute to level 4 autonomous vehicle systems include perception, planning and control. The adoption of state-of-the-art sensors like Lidar, cameras, radar, Global Navigation and Satellite System (GNSS) in the perception module helps in utilizing the information about the surrounding environment. The planning module devises the path planning, obstacle avoidance and behaviour planning for the autonomous vehicle. Finally, the control module governs the course of action using longitudinal and lateral controllers.

**Figure 1:**
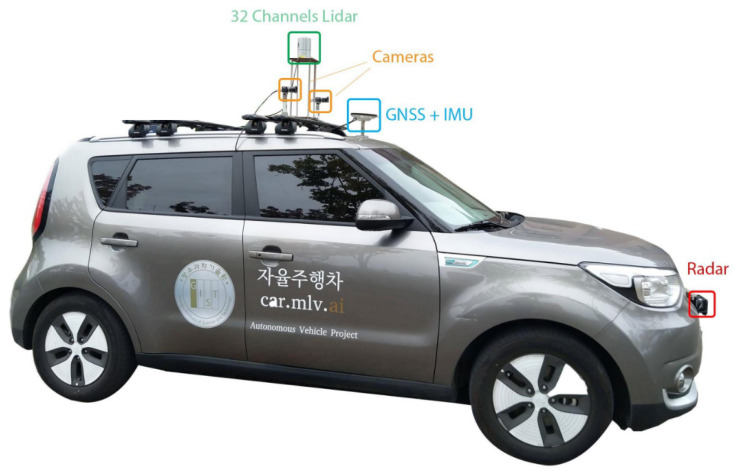
Our KIA-Soul EV is equipped with two FLIR cameras, a 3D 32-laser scanner and a GPS/IMU inertial navigation system [24] (Best viewed in color).

In this study, we have developed an autonomous vehicle using limited sensor suite as compared to autonomous vehicles discussed previously. Figure 1 illustrates our test-bed called Car.Mlv.ai. The link (https://goo.gl/KdUyzA) provides the demonstration videos of our autonomous vehicle. The efficacy of our autonomous vehicle is experimentally verified by deploying it as an automated taxi service in the constrained environment. The proposed autonomous vehicle is composed of localization, perception, planning and control modules. The design of a distributed system and incorporation of robust algorithms enable the autonomous vehicle to perform efficiently. The fusion of sensor data for localization in map generation and navigation and also in perception module enable reliable object detection, recognition and classification in a dynamic environment. In the planning module, the optimal path is devised by considering the lane, obstacle information, and upon which velocity and behaviour planning are executed. Finally, based on the planning results, the control module performs the lateral and longitudinal control of the autonomous vehicle. In this work, the main contributions are as follows:

(1) The proposed autonomous vehicle (car.Mlv.ai) is designed with constraint resources with minimal sensor suite compared to state-of-the-art vehicles. The cost-effectiveness of the proposed autonomous vehicle is one of the significant contributions of the proposed work.
(2) The generation of 3D map for the localization of autonomous vehicle is built using the 32 channels Lidar with the auxiliary information for Global Navigation Satellite System (GNSS), Inertial Measurement Unit (IMU), and odometry data from vehicle Controller Area Network (CAN) bus respectively.
(3) For the autonomous vehicle’s perception, the fusion of exteroceptive sensors that include Lidar and camera is performed in the proposed work. In the fusion framework, both sensor’s object detection networks are trained and implemented in the Robot Operating System (ROS). The real-time efficacy of the detection network is evaluated by using TensorRT and deployed on the system.
(4) A state-machine is designed that caters the mission and motion planning for the autonomous vehicle by incorporating the information of obstacles from the perception stack.
(5) For longitudinal and lateral motion control, a customized controller, named Kiasoul-Socket is designed that caters throttle, brake, and steering of the autonomous vehicle by following the state-machine conditions for different real-world traffic scenarios, for instance, obstacle avoidance and obstacle stopping. The KIA Soul EV company does not provide this controller because of proprietary law.
(6) Development of CAN-BUS shield for KIA Soul EV for acquiring the CAN messages from the vehicle for lateral and longitudinal control and providing the odometry data for the localization.
(7) The proposed autonomous vehicle has experimentally validated in the application of autonomous taxi service.

The remainder of the paper is organized as follows—Section 2 explains the platform for the autonomous vehicle that includes the overview of the platform along with sensors information and their installation on the autonomous vehicle. Section 3 focuses on the architecture of the autonomous vehicle in which all the four modules of the autonomous vehicle are discussed. The experimental validation in the context of autonomous taxi service is discussed in Section 4. The comparison of proposed autonomous vehicle with state-of-the-art is discussed in Section 5. Section 6 discuss the challenges faced in developing the autonomous vehicle, and finally, Section 7 concludes the paper.

## 2. Autonomous Vehicle Platform

Autonomous driving systems are equipped with state-of-the-art sensors for robustness and reliability. In this work, we have used KIA Soul EV and retrofitted it by installing the exteroceptive and proprioceptive sensor for perception, navigation and internal vehicle monitoring tasks as shown in Figure 1.

Exteroceptive sensors are used for the perception of the environment. The most common modalities that are used for the perception are camera and Lidar. In designing the autonomous vehicle, two FLIR Blackfly cameras along with one Velodyne Lidar have used for perceiving the environment. Figure 1 shows the design mount for Lidar and camera. The cameras are operating at 196 frame rate giving 1600×1100 resolution of images. The Velodyne Lidar has 32 channels giving up to 695,000 points per second having a range of up to 100 m with the tolerance of ±2 m. In the integration of cameras and Lidar to the autonomous vehicle, installation considerations are kept in view to avoid the calibration misalignment between the camera, and Lidar. The camera is directly placed under the zero axis of the Lidar. The Lidar height is selected empirically such that field of view does not include the car bonnet and trunk.

The precise inertial navigation of the autonomous vehicle is achieved by the integration of proprioceptive sensor in the sensors stack of the autonomous vehicle. For this purpose, the Novatel Global Navigation Satellite System (GNSS) is used which is a single device enclosing Global Positioning System (GPS) and Inertial Navigation System (INS) having an accuracy up to ±2 cm. The Novatel module contains inertial measurement unit (IMU) component, which consists of Fiber Optic Gyros (FOG) and Micro-Electromechanical System (MEMS) accelerometers. In addition, the odometry data is read from the vehicle Controller-Area-Network (CAN) bus. A CAN-BUS shield is used for reading the data from the CAN bus through OBD (On-board Diagnostic)-II connector. CAN-BUS shield is an MCP2515 CAN bus controller with SPI interface. It gives the data at a rate of 1 Mb/s.

**Figure 2:**
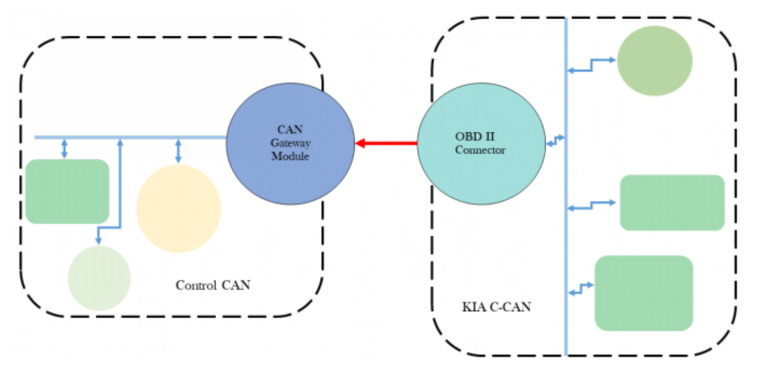
The communication lines between CAN Gateways Module and OBD Connector.

The design and development of control for an autonomous vehicle constitute towards the safe manoeuvring. In this respect, a drivekit which is an electronic controller specifically designed for drive-by-wire cars is used. It provides precise control by making an abstract layer on the top of the vehicle CAN bus, yet not affecting the vehicle own CAN bus. The drivekit provides its own CAN bus called Control-CAN which communicates with the vehicle CAN bus through a CAN Gateway for transferring and receiving the control signals. Figure 2 shows the communication between Control-CAN and vehicle CAN bus. Further, a specified controller Kiasoul-socket is designed for lateral and longitudinal control that incorporates CAN and planning data. The Kiasoul-socket generates the control command for each control signal, in this case, steering, braking, and throttle. The generated control command is fed to the actuators of the autonomous vehicle through the drivekit.

The modular approach is adapted to efficiently cater the computing consideration by using one server and laptop. In addition, two embedded systems which include raspberry Pi are also integrated into the computation stack. All the communication between the computers is carried over a gigabit Ethernet link with ROS running on the laptop and server. The vision sensors data is parsed to the server for the perception and planning of the environment while the Lidar and proprioceptive sensors data is processed by the laptop. The Kiasoul-socket controller is developed on one Raspberry Pi that generates the control command and communicates with the drivekit. The other Raspberry Pi is used for getting the GNSS data and then publish the data to be used by the laptop for localization and navigation purposes. The server acts as a ROS master, and all other other computing units communicate with it.

**Figure 3:**
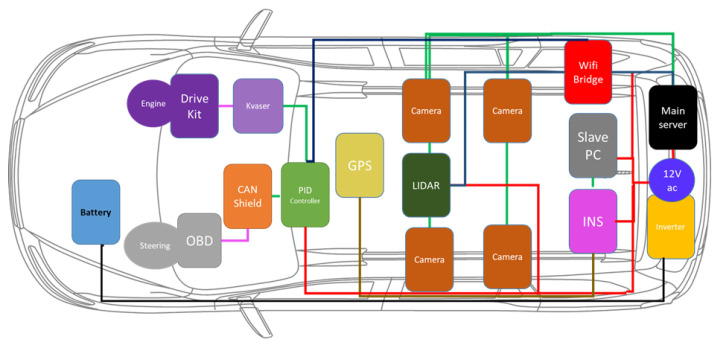
In the wiring diagram, the black color is the connection between the main battery of the car and the inverter. The red color wire shows the power is transmitted to different sensors. The green colors show the connection between sensors which are connected to the computer and are powered through them. The violet color is for the devices which are connected through a network protocol. The brown color is for GPS antenna of the GNSS system. The pink color is for receiving and sending commands to car hardware (Best viewed in color).

Figure 3 shows the wiring of power utilization between all computing and communication components. KIA Soul EV is equipped with 360 *V* DC, lithium-ion polymer battery having the capacity of 27 kW h and power of 90 kW. All the sensors, computing units, and gigabit Ethernet module require AC power, so in order to meet that demand of AC power, an inverter is placed in the trunk of the car which provides necessary AC power to all equipment.

## 3. Architecture

**Figure 4:**
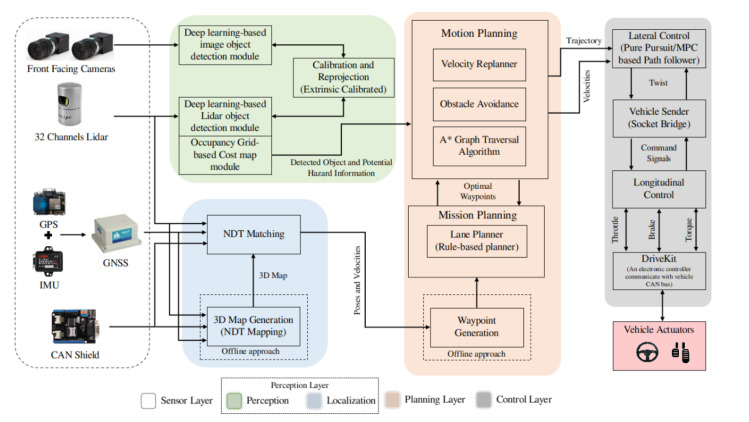
Overall architecture of our autonomous vehicle system. It includes sensors, perception, planning and control modules (Best viewed in color).

The architecture of the autonomous vehicle is composed of four major layers, as illustrated in Figure 4, that are sensor layer, perception layer, planning layer and control layer. The sensor layer constitutes of exteroceptive and proprioceptive sensor modalities which provide the data to the different layer’s modules. In the perception layer, the two main elements that contribute toward the environment understanding are detection and localization. The understanding of the environment in the perception layer provides the necessary information to the planning layer. The planning layer devises the motion, mission and trajectory planning of the autonomous vehicle based on the observation accumulated in the perception layer. The decision from the planning layer is fed to the control layer for the execution of the control command to vehicle actuators through the lateral and longitudinal controller. The following subsections describe the modules used in perception, planning and control layers for the autonomous vehicle.

### 3.1. Mapping and Localization

Localization is the foremost and essential task for the autonomy of the autonomous vehicle. Localization is the task of determining ego-position relative to the frame of reference in the dynamic environment. It facilitates any mobile robot to navigate the environment and avoid dangerous circumstances such as obstacles, collisions, and unsafe states. Typically, localization can be achieved using three techniques—utilizing GPS and IMU data, SLAM (Simultaneous Localization And Mapping), and Priori map-based localization. Here, we have implemented Priori map-based localization on the core concept of correlation [25]. It achieves localization by correlating the online data received to offline map built prior and finds the best-matched location. However, matching is not always optimal; therefore, data from GPS and IMU are used to correct accumulated errors.

In the context of autonomous vehicles, state-of-the-art localization systems utilize multi-modal point cloud matching techniques. The online point cloud obtained, which represents the environment’s situation, is matched against the prior point cloud map. The online scan is rotated and translated around its center to obtain position and orientation that gives a strong correlation between the two point clouds. The localized position of the sensor relative to the map gives the pose of the vehicle. The GPS and IMU data are used along with dead reckoning. The initial pose is estimated from GPS data. In summary, three main methods exist that use point cloud for mapping and localization. These methods include Normal Distribution Transform (NDT) [26], Iterative Closest Matching (ICP) [27], and Simultaneous localization and matching (SLAM) [28].

SLAM algorithm maps the environment by detecting similar features in two-point clouds and minimize their distance. It also detects loop closure [29]. Kim et al. have proposed 2D hector SLAM that generates the environment map in real-time [30], but the problem with SLAM is drift, which is caused by the accumulation of error in estimating the pose. Besl et al. have proposed the initial iterative closest matching (ICP) algorithm for scan matching by using that initial model [31]. He et al. have presented the modified version of ICP for registering 3D point cloud data [32] by using the geometric features of point cloud data. The main drawback of using ICP is its sensitivity to initialization.

Biber et al. have introduced Normal distribution transform for matching a 2D laser scan [33]. It transforms the points in the scan into a piece-wise continuous and differentiable probability density. The probability density comprises of a set of normal distributions. In order to match two laser scans, the Normal distribution transform sum is maximized. Various modified variants of NDT have proposed by substituting the minimization function. Prieto et al. have used NDT with the differential evolution to reduce the error between the NDT of two-point clouds [34]. Zhou et al. have written a modern 3D library for processing point cloud [35].

**Figure 5:**
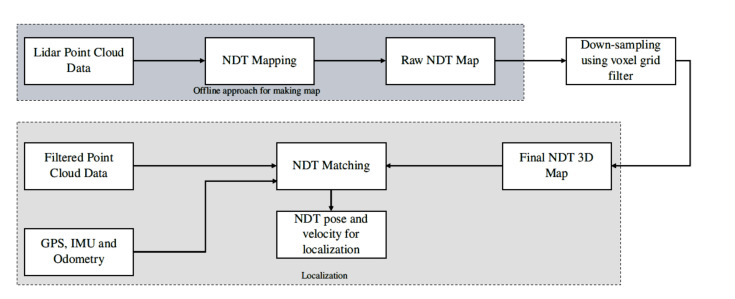
The flow diagram of the localization process is shown. First, the map is built using NDT mapping using the Lidar data. The point cloud data is downsampled using voxel grid filter for the refinement of 3D map. NDT matching takes filtered Lidar data (scans), GPS, IMU, Odometry, and 3D map for the pose estimation.

Here, we employ the 3D normal distribution transform for mapping and localization using point cloud [36]. Figure 5 shows the overall framework for the generation of 3D point cloud map and localization of autonomous vehicle. The NDT of the reference point cloud is determined first for the generation of the map that results in ND voxels. In order to apply the NDT on the input point cloud data, its initial position is determined using odometry data and converted into ND voxels. The two voxels correlate and registered by taking average and covariance. In order to build the map, an estimation step is performed, and if the parameters converge, the input point cloud is combined with the reference point cloud [37].

For localization in the current environment, NDT matching is used. The matching of scan point cloud data with 3D map using NDT is a search problem. Equation (1) is used to transform the input point cloud to match the 3D point cloud. The optimal parameter *t* is found by correlating the input point cloud data, and the reference point cloud data.

(1) $$\begin{array}{c} x_{i}^{{}^{\prime }}=Rx_{i}+t^{i}, \end{array}$$

where, *R* is the rotation matrix and $t=[\alpha ,\beta ,\gamma ,t_{x},t_{y},t_{z}]$ are Euler angles and translation parameters. The efficacy of this transformation is evaluated using the fitness score computed by Newton non-linear optimization, as shown in Equation (2).

(2) $$E\left(X,t\right)=\sum_{i=1}^{N-1}\exp\frac{-{\left(x_{i}^{{}^{\prime }}-p_{i}\right)}^{t}\sum_{i}^{-1}\left(x_{i}^{{}^{\prime }}-p_{i}\right)}{2}.$$

#### Experimental Results

**Figure 6:**
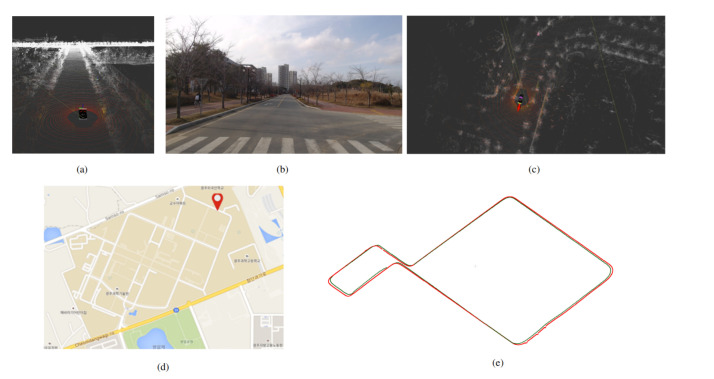
(**a**) The 3D map of the environment formed using the NDT mapping. (**b**) The localization of autonomous vehicle using NDT matching in the 3D map by matching the current Lidar scan and the 3D map information. (**c**) The current view of the environment in image data. (**d**) The current localization is marked in the Google map. (**e**) Qualitative comparison between GNSS pose and NDT pose. The GNSS pose is shown in red and NDT pose is shown in green (Best viewed in color).

To summaries and evaluate, we have implemented NDT mapping and localization using the point cloud. The parameters for NDT matching, as illustrated in Table 1. In Table 1, the Error Threshold represents the amount of error it can accumulate in matching the current scan with the 3D map. The Transformation Epsilon illustrates the maximum difference between two consecutive transformations to be optimized for convergence. The Max iteration is the number of iteration required to calculate the alignment. The localization is done in real-time, as shown in Figure 5. It takes filtered Lidar point cloud data, GPS, IMU, and odometry data. The IMU and odometry data reduces the error by NDT matching and reduce the rate of false NDT matching. The outcome of localization using NDT matching is shown in Figure 6. The efficacy of NDT matching is computed by making quantitative and qualitative comparisons. For the quantitative analysis, root mean squared error (RMSE) is computed between the GNSS pose and NDT pose. In this regard, the GNSS pose is the ground-truth that is obtained from the GNSS sensor. The RMSE is computed in *x*, *y*, and *z* directions along with the overall RMSE is also computed. Table 2 shows the quantitative comparison between GNSS and NDT pose. As the range of data is in 10,000, the values of RMSE in each case are relatively small, showing better accuracy. Figure 6e shows the qualitative comparison of NDT and GNSS pose of the route for which the autonomous car is being driven. It can be seen that minimal error exists between NDT pose and GNSS pose.

### 3.2. Perception

**Figure 7:**
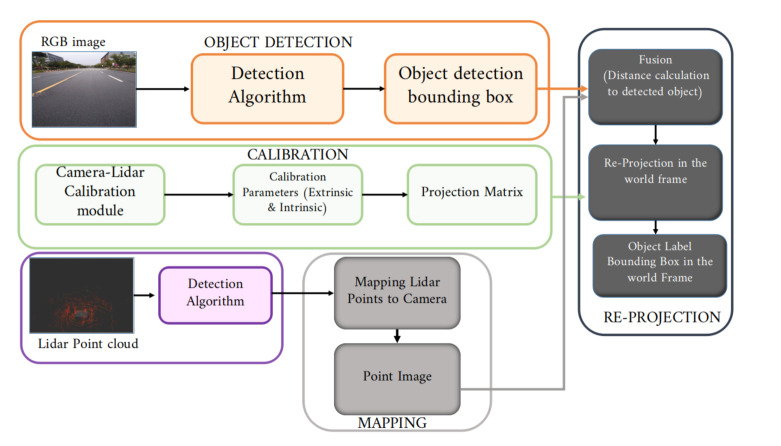
The overall architecture of the calibration and re-projection process is shown. The process is divided into steps. (i) Object detection gives the object detection (ii) Calibration module (iii) The calibration parameters are utilized by the mapping module for generating the point image. (iv) The re-projection module consists of range fusion that performs the distance calculation from the point image and object detection proposal in images and finally computes the object labels bounding box in the Lidar frame (Best viewed in color).

*‘What’s around me’* advocates that the operation of the autonomous vehicle, demands in-depth understanding and analysis of the environment, to take the decisions for the safe navigation. For instance, at the crosswalk, the precise locations of a pedestrian and a bicyclist will allow the autonomous vehicle to make an imperative decision. For perceiving the environment, in this work, we have utilized two sensor modalities—Lidar and cameras, for object detection and recognition tasks [38]. The data fusion of sensor modalities contribute to the reliability of perception of the environment and also surmount the hardware constraints of sensors. Figure 7 illustrates the perception framework for the autonomous vehicle that involves three main steps (i) object detection using camera and Lidar, (ii) camera-Lidar calibration and (iii) fusion (mapping and reprojection). The remainder of this section explains the three tasks for the perception of the autonomous vehicle.

#### 3.2.1. Object Detection

The core idea of perception is to determine the location and size of the object of interest in the dynamic environment for the autonomous vehicle. Object detection enhances the perception framework by identifying and recognizing different traffic entities (pedestrians, vehicles or cyclist) using vision camera and Lidar as sensor modalities. In the context of image-based object detection, the generalized object detection is evolved tremendously by consolidating the deep learning techniques. Moreover, the advancement in the computation hardware also makes it possible for the image-based deep learning object detection algorithms to be deployed in the autonomous driving perception framework. Besides, the increase in computing power, the image-based deep learning object detection algorithms must be in real-time and reliable for autonomous driving. For this purpose, we have used, the perception framework using single-stage object detection network rather than the region proposal detection framework. In this perception framework, YOLOv3 (You Only Look Once) [39] is used for object detection using camera data. YOLOv3 is a single-stage object detection framework that using deep convolutional neural network for extracting the features by segregating the input image into grids. The class probabilities and the bounding box parameters are predicted by the use of a fully connected layer.

The affordability and availability of the camera makes it an elementary sensor modality for the perception tasks, but it has a drawback with illumination variation that affect the performance of object detection algorithms. In addition to illumination variation, the camera-based object detection operates in the projected image space in which it is difficult to estimate the scale of the scene. In order to overcome this hindrance, Lidar based object detection is used to bridge the gap between 2D image-based detection and project the outcome of the detection in the 3D frame. In literature, much work has been carried out to use Lidar data for the detection and classification of the object. Azam et al. have used multiple frames of Lidar to generate the CAD models, and then employs the convolution neural network architecture for the detection and classification of point cloud data [40,41,42,43]. Bo Li et al. have used a convolution neural network for the object detection and classification in point cloud data [44]. Chen et al. have made a two-network architecture: one for 3D object proposal generation and one for the multi-view feature extraction [45]. They have designed a fusion mechanism for detecting the 3D object using these two networks. Martin et al. have used the voting scheme for object detection by exploiting the sparsity of the point cloud data using a convolution neural network [46]. In accordance with Lidar-based object detection, we have opted the PointPillar for 3D object detection [47].

For the 3D object detection, the point cloud is filtered first by removing the ground plane before passing it to PointPillar network. The ground plane is removed by fitting a plane and applying a two-step filter. This filter uses angle and distance of two consecutive points to differentiate the ground points from vertical points. Algorithm 1 shows the pseudo-code for angle and distance-based filtering. The PointPillar network is composed of three main sub-modules (i) Pillar Feature Network, (ii) Backbone, and (iii) SSD Detection head. The filtered point cloud data is converted to pseudo-image for feature extraction by Pillar Feature network, which acts an encoder. The Backbone network learns the features of pseudo-image using a convolutional neural network, and finally, the SSD Detection Head is utilized to perform the 3D object detection. In addition, a unified Interacting-Multiple-Model with Unscented Kalman Filter and Joint Probability Data Association Filter (IMM-UK-JPDAF) tracker is used for tracking the 3D object detection [48].
**Algorithm 1:** Ground plane removal based on angle and distance filtering
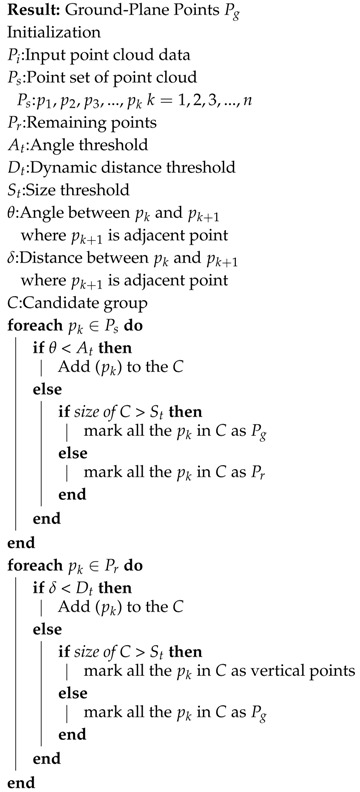


#### 3.2.2. Camera-Lidar Calibration

In the literature, researchers have used the calibration process for solving computer vision and robotics problems. In this work, we have used the camera-Lidar calibration as a bridge to project the 2D object detection results on the 3D frame. The camera-Lidar calibration has been divided into three main categories depending on the use of calibration objects—planar chessboard [49,50], different forms of planar surface [51,52] and no calibration object [53,54]. Similarly, some works have also been done by auto-calibration of camera and Lidar [55,56,57,58,59].

The whole process of camera-Lidar calibration constitutes of three steps (i) Grabbing both sensor data, (ii) Extracting the points on the chessboard and (iii) Calibration optimization. First, the data is collected for 30 different poses of checkerboard by placing it in the range of both sensor’s field of view. In images, the checkerboard is extracted using OpenCV corner detection algorithm. For Lidar, the points are manually selected, that corresponds to the checkerboard location. The optimization is done on the camera intrinsic and extrinsic calibration parameters. The intrinsic camera calibration, which gives the position and orientation of each grabbed chessboard in the camera, are evaluated by the OpenCV PnP method [60]. The extrinsic calibration parameters involve the computation of the rotation matrix first followed by optimizing the translations as illustrated in Equations (3) and (4).

(3) $$\begin{array}{cc} \; & R^{*}\cdot N=M,\\ \; & R^{*}\cdot \left(N\cdot N^{\prime }\right)=M\cdot N^{\prime }\\ \; & R^{*}=\left(M\cdot N^{\prime }\right)\cdot {\left(N\cdot N^{\prime }\right)}^{-}1 \end{array}$$

(4) $$\begin{array}{c} {\left(x,y,z\right)}^{*}=\arg\min_{x,y,z}\sum_{i}\sum_{j}{\left({\left(R^{*}\cdot p_{i}+T\left(x,y,z\right)-q_{i,j}\right)}^{\prime }\cdot \left(R^{*}\cdot n_{i}\right)\right)}^{2}, \end{array}$$

where *R* is the rotation matrix, *N* is matrix formed by stacking all normals of grabbed chessboard in camera coordinate and *M* is the normal matrix grabbed in Lidar coordinate. T(x,y,z) is translation matrix, $q_{i,j}$ is the $i_{th}$ chessboard’s $j_{th}$ point in the Lidar coordinate, $p_{i}$ is the $i_{th}$ chessboard’s position in camera coordinate frame and $n_{i}$ is chessboard’s normal vector in camera coordinate frame.

#### 3.2.3. Fusion (Mapping and Re-Projection)

The object detection in both sensor modalities and camera-Lidar calibration provides the foundation for the fusion of both sensor data. The fusion of both sensor modalities is carried out using the object detection matching. First, the point image is created by mapping the 3D detected object 2D frame. This point image, along with image-object detection bounding box and camera-Lidar calibration parameters are fed to reprojection module. In reprojection module, the mapping of 3D to 2D is evaluated and considered it a match if the overlapping of 3D object projection over the 2D detected object is greater than 50%. The corresponding 2D object label is attached to the corresponding 3D object and projected in the 3D frame.

#### 3.2.4. Experimental Results

**Figure 8:**
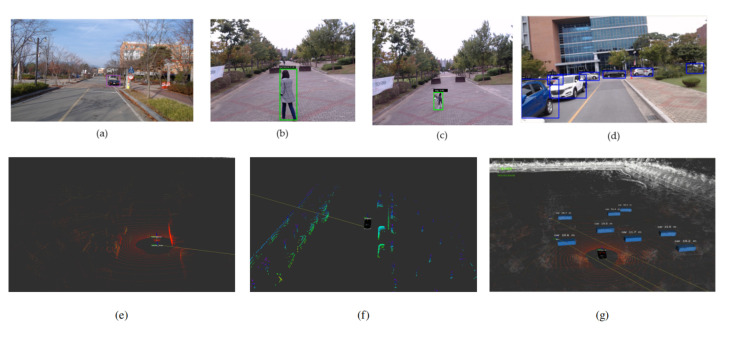
(**a**–**d**) The object detection result using YOLOv3. (**e**) shows lidar point cloud, (**f**) shows the results for ground plane removal and (**g**) shows the results of PointPillar (Best viewed in color).

**Figure 9:**
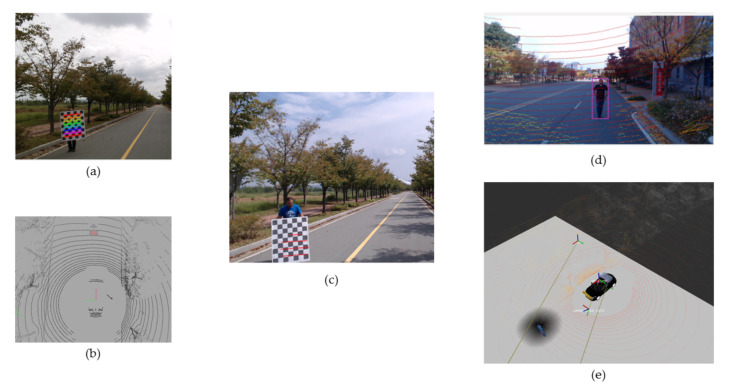
(**a**,**b**) The images data and Lidar point cloud data. (**a**) The corner detection in image data. (**c**) The point image data is the projection of Lidar data onto image data. (**d**) The person is detected in the image data and (**e**) The re-projection of that detection in the Lidar data (Best viewed in color).

The experimental validation of our perception module is performed by implementing the whole stack in the ROS environment. The camera-based object detection is performed in the camera frame where YOLOv3 is implemented as ROS node and trained on the COCO dataset having a learning rate of 0.001. Figure 8a–c shows the qualitative detection results of YOLOv3. In the context of Lidar-based object detection, first, the ground plane is removed using a two-step filter. Table 3 shows parameters for ground plane removal. The Sensor height (in meters) corresponds to the Lidar sensor’s installation on the proposed autonomous vehicle above the ground. The Distance threshold (in meters) illustrates the minimum distance for the two consecutive points included in the vertical points rather than ground points. The Angle threshold (in radians) constitutes the inclusion of points in the candidate group. The size threshold (number of points) determines the size of the candidate group. Figure 8f illustrates the ground plane removed point cloud. This ground plane removed point cloud is fed to PointPillar (implemented as ROS node) for the object detection in the Lidar frame [47]. The PointPillar network is first trained on the Kitti dataset [19]. Figure 8g shows the detection results in the Lidar frame. The camera-Lidar calibration bridges the camera and Lidar frames. The outcome of camera-Lidar calibration is depicted in Figure 9.

### 3.3. Planning

**Figure 10:**
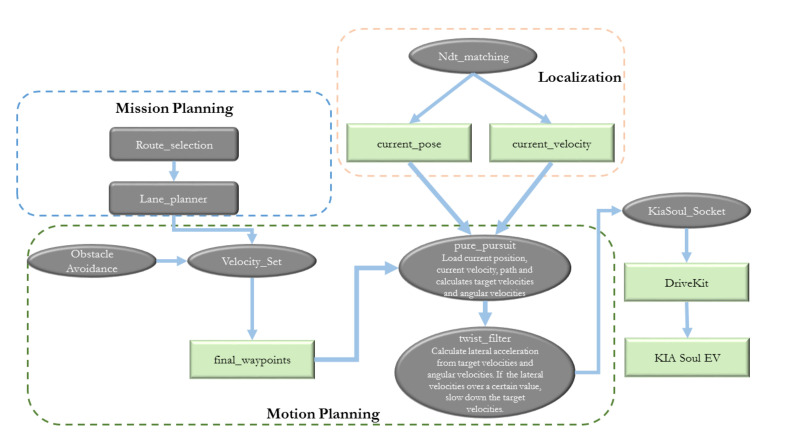
The architecture of the planning module is shown. The planning module contains Mission and Motion planning as its most vital modules. Mission planning generates the lane for routing for the autonomous vehicle using lane planner. Motion planning plans the path and keeps the autonomous vehicle to follow that path. The flow of information is shown above (Best viewed in color).

Human driving addresses the problem of realistic traffic scenario and makes an optimal decision based on the current predicament because of traffic congestion or unwanted circumstances. An autonomous vehicle while operating in these scenarios must take an indispensable decision for which the planning module comes into play. The planning module of our autonomous vehicle integrates mission and motion planning for optimal trajectory generation, and behavior planning. Figure 10 shows the details of the planning module.

#### 3.3.1. Path Generation

**Figure 11:**
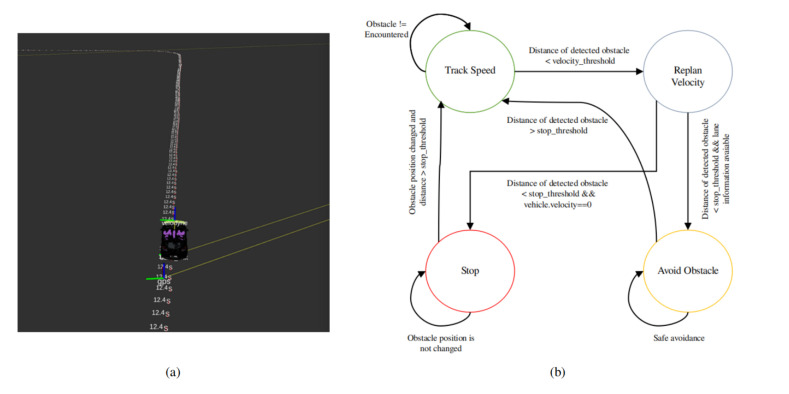
(**a**) The waypoints generated by the motion planning module that contains the GPS coordinates, velocities and angles. (**b**) The state-machine implementation for obstacle avoidance and stopping. The vehicle will approach to track the speed until the obstacle is not encountered. When the obstacle is detected, if the distance of detected obstacle is less than the velocity-threshold, the planner will replan the velocity to decrease the speed. After this there are two options for the vehicle, if the distance of the detected object is less than stop-threshold and lane information with change flag is available, then the vehicle will avoid the obstacle until the safe avoidance has not been done. In another case, if the detected obstacle distance is less than stop-threshold, and vehicle velocity is approaching to zero, the vehicle will stop until the obstacle position is not changed. Finally, suppose the obstacle position is changed, and the detected obstacle distance is greater than the stop-threshold. In that case, the vehicle will approach to track speed in stop case whereas in avoiding obstacle case if the distance of detected obstacle is greater than stop-threshold, it will approach to track speed (Best viewed in color).

The path is generated using the waypoints which are composed of pose and velocities obtained from the localization. These waypoints serve as direction to follow for the autonomous vehicle. These waypoints are processed by the lane planner (mission planning) and depending on the traffic conditions; these waypoints are manipulated. Figure 11a shows the waypoints generated and followed by the autonomous vehicle.

#### 3.3.2. Mission Planning

The mission planning of the autonomous vehicle is designed as a rule-based planner that incorporates the lane information. The lane planner receives multiple routes in the form of waypoints (pose, velocities, yaw, number of lanes and change flag) and using the lane information it calculates the nearest path from the current location. Based on the current route, it detects the left and right lane using the lane change flag. The optimal route is selected for the autonomous vehicle and published as optimal waypoints for the motion planning for path planning.

#### 3.3.3. Motion Planning

The motion planning module deals with path generation and path planning by incorporating the information of lane, obstacle detection, and velocities.

##### Path Planning

Planner for the planning can be grouped into the global planner and local planner. The global planner servers as the path to be followed by the autonomous vehicle over the entire coarse. The local planner comprises of trajectory planning and behaviour planning in the form of obstacle avoidance/stopping and velocity replanning.

Smooth trajectory planning and obstacle avoidance are the primary purpose of path planning and can be consolidated in the local planner. In the context of path planning for the autonomous vehicle, the local planner uses the modified A-star (A*) graph traversal algorithm for the path-finding with the addition of obstacle avoidance and velocity replanning [61]. In our case, the lane planner serves as a global planner because it is based on waypoints.

**Figure 12:**
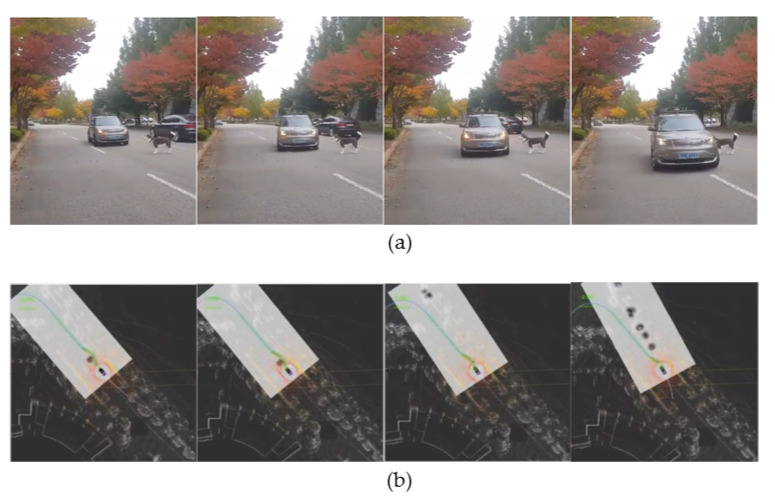
(**a**) The frames in which the obstacle avoidance is done. An obstacle (a mannequin husky) is placed in front of the autonomous vehicle. (**b**) The visualization of pure pursuit and the changed waypoints path (Best viewed in color).

**Figure 13:**
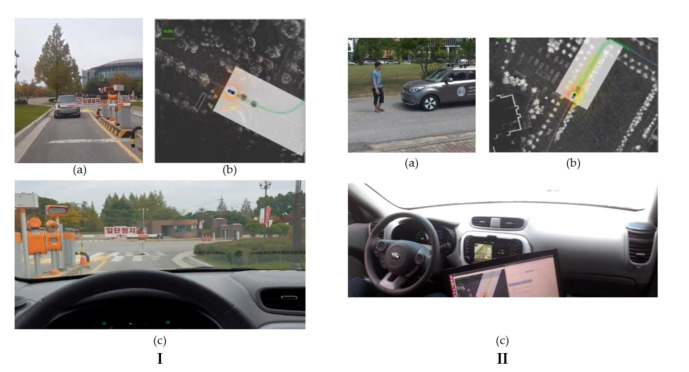
(**I**) (**a**,**c**) The autonomous vehicle is stopped in front of the obstacle. (**b**) The visualization of the process in the RViz showing the obstacle and the car. (**II**) (**a**–**c**) shows the results of an obstacle stop using the person as an obstacle (Best viewed in color).

The obstacle avoidance and velocity replanning are implemented as a state-machine by incorporating the information from perception module and lane planner. Figure 11b shows the state-machine for obstacle avoidance and velocity replanning. The obstacle avoidance receives the input from the perception module and depending on the presence of an obstacle, and by complying the traffic rules for two-way single lane and multiple lanes; it will avoid the obstacle by changing the waypoints with the desired velocity that is managed by the velocity module. When an object is detected, the autonomous vehicle has two options depending upon the environment: it can stop and wait for the obstacle to move or avoid the obstacle. If the obstacle is detected in the two-way single lane environment, the autonomous vehicle will stop complying to traffic rules, and in later case of multiple lane environment it will avoid the obstacle. The decision to obstacle avoidance and stopping is based on the distance from obstacle and lane information. The distance between the autonomous vehicle and obstacle is divided into two regions (i) velocity-replanning distance to slow down the vehicle and (ii) stop distance. The optimal distance for both region is 10 m each, and this is given as a parameter to the local planner. Figure 12 shows the obstacle avoidance and Figure 13 shows the car stopping for the obstacle until it is removed.

#### 3.3.4. Experimental Results

The planning module of our autonomous vehicle integrates mission and motion planning. The autonomous vehicle’s mission planning is designed as a rule-based planner that incorporates the information of the lane. The motion planning is implemented as a state-machine employing obstacle avoidance and velocity replanning. The Table 4 illustrates the parameters for the planning module. The velocity replanning distance threshold (in meters) and stop distance for obstacle threshold (in meters) corresponds to the region distance upon the vehicle will slow down and stop upon the detection of the obstacle. The detection range (in meters) shows the detection area on the proposed autonomous vehicle route. The threshold for objects determines the number of points that can be considered as an obstacle. The deceleration upon detection of the obstacle is determined by deceleration for obstacle (m/s${}^{2}$). The curve angle (in radians) determines the angle the proposed vehicle will follow on the obstacle avoidance.

**Figure 14:**
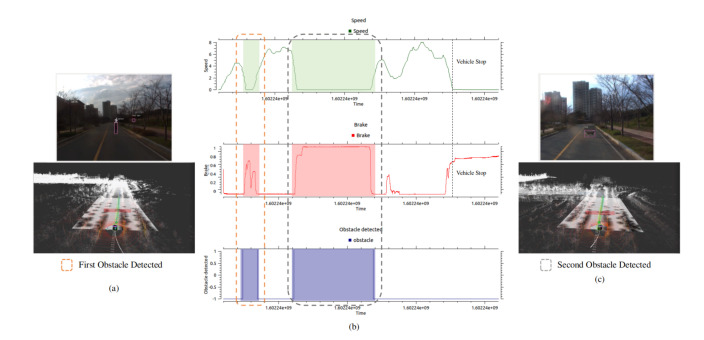
The quantitative evaluation of obstacle detection using the proposed autonomous vehicle is illustrated. (**a**,**c**) show the obstacle detection in camera and Lidar frame using YOLOv3 and PointPillar network respectively. (**b**) The latency of speed, brake and obstacle profile is illustrated. It is shown in the graph that when the obstacle is detected, the vehicle speed slows down and brake command is promptly activated. The synchronization between the three profile is clearly seen with total execution time of 1650 μs. In (**b**) at the detection of second obstacle, the graph profile of speed, brake and obstacle remain in their respective state, this corresponds that the obstacle is present in the route of autonomous vehicle and have not changed the place for that much period. The speed is in (m/s), brake command is command to vehicle CAN bus through drivekit, and obstacle detection is the indication of obstacle flag.

**Figure 15:**
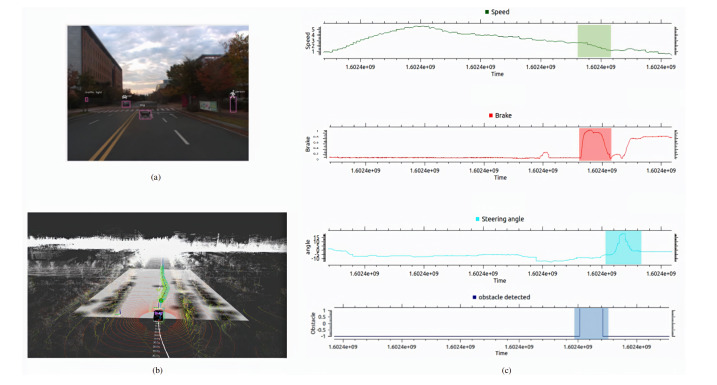
The quantitative evaluation of obstacle avoidance using the proposed autonomous vehicle is illustrated. (**a**) Obstacle is detected in the camera frame using YOLOv3. (**b**) Obstacle is detected in the Lidar frame using PointPillar and upon detection the waypoints are changed and velocity is replanned using velocity replanning module and obstacle is avoided by complying the traffic rules using the state-machine configuration. (**c**) illustrates the quantitative graphs of obstacle avoidance using proposed autonomous vehicle. The spike in the steering angle graph shows the change of angle in positive direction. In the graphs it is shown that first the obstacle is detected, the vehicle speed is lower down and brake is applied, and in that case it is possible for the autonomous vehicle to avoid the obstacle by not violating the traffic rules, the autonomous vehicle has avoided the obstacle safely.

We have conducted the experimentation to illustrate the quantitative evaluation of proposed autonomous vehicle. In the first experimentation we have provided the quantitative evaluation of vehicle stopping upon the detection of obstacle. Figure 14 shows the experimental results of obstacle detection. The quantitative graphs in Figure 14 illustrate that when the obstacle is detected by the vehicle, the brake command is active and the vehicle speed is slowed down. For the obstacle avoidance we have conducted the same experiment and the Figure 15 shows the quantitative results.

### 3.4. Control

’What should I do?’ acknowledge the autonomous vehicle’s concern to execute the commands received from the planning module by providing the necessary inputs to the actuators that will generate the desired motions. The planning layer generates the velocity and trajectory profile which autonomous vehicle requires to follow. The controller for the autonomous vehicle is designed by keeping in view the safety and stability concerns. In the context of the autonomous vehicle, modelling of vehicle dynamics plays an essential part in selecting and designing the optimal controller. The vehicle dynamics model constitutes the longitudinal and lateral model of vehicle [62]. These vehicle models define the complete motion model and can be used for designing the controller. The vehicle’s longitudinal model is based on the dynamics of the model that generates the forward motion. The lateral model of vehicle dynamics constitutes towards the modelling of smooth manoeuvring of the vehicle.

#### 3.4.1. Longitudinal Control

The generation of velocity profile for the autonomous vehicle is performed by the longitudinal control. In classical control, feedback controllers are used in several applications because they are actively compensating for the deviation between the measured and desired responses. Proportional-Integral-Derivative (PID) is the feedback controller that is extensively used in the process control industry [63,64]. The PID controller relies on the propagation of error and minimizes it to deliver the desired response of the system [65]. Equation (5) shows the control law of PID, which is based on the error signal

(5) $$\begin{array}{cc} u\left(t\right)=k_{d}\dot{e}+k_{p}e+k_{i}\int edt, & \end{array}$$

where, $k_{p}$, $k_{i}$, and $k_{d}$ are the proportional, integral and derivative constant of the controller respectively, and *e* corresponds to the error signal. In our case, for the autonomous vehicle, the velocity profile of longitudinal control constitutes of brake, torque, and throttle. For the vehicle to follow the velocity profile, a customized PID controller is designed to minimize the difference between current and target values of the torque, brake, and throttle. The current values are obtained from the vehicle CAN bus, whereas the target values are taken from the planning layer. The proprietary laws of KIA Company do not share the CAN bus IDs, and the CAN bus IDs are empirically found using CAN sniffer (https://github.com/linux-can/can-utils). For each throttle, torque and brake a PID controller is designed with two sets of control parameters. This framework provides flexibility to control the vehicle on high and low torque for the steering wheel, and also the high and low speed for throttle/brake.

**Figure 16:**
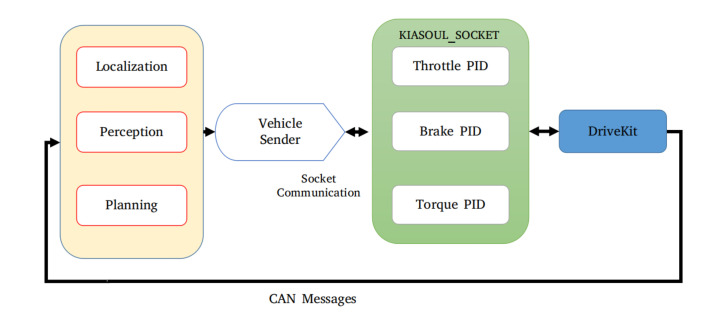
Vehicle sender receives the information from Planning and send the command to PID controller. Kiasoul-socket is composed of Throttle/Brake and Torque PIDs that sends the command to Drivekit.

The framework for the PID controller is shown in Figure 16, which include (i) Vehicle sender (ii) PID controller (customized), and (iii) Actuator. The purpose of vehicle sender is to provide a communication bridge between software and hardware stacks, and sends commands over the socket to the customized PID controller.

#### 3.4.2. Lateral Control

The path tracking for the autonomous vehicle incorporates the design of lateral control. In our case, two path tracking algorithms are implemented for the lateral control, (i) Pure Pursuit, and (ii) Model Predictive Control (MPC)-based path follower.

**Figure 17:**
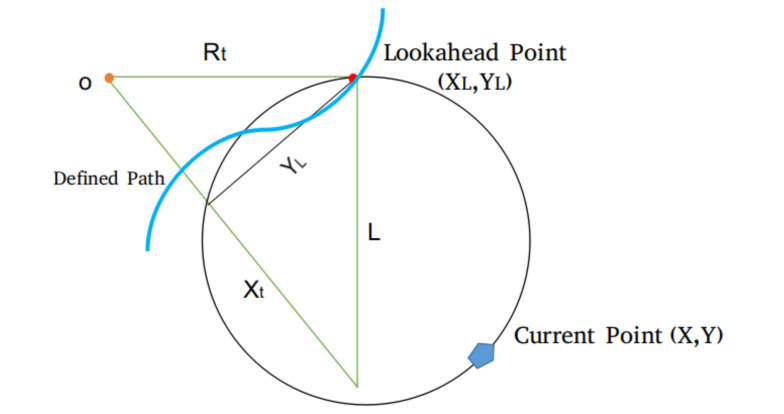
The geometric representation of pure pursuit algorithm.

Pure Pursuit is a path tracking algorithm which estimates the curvature from the current location to the goal location. The goal position is re-calculated such that it is constant distance ahead of the current vehicle location. In summary, Pure Pursuit chases a changing goal position some distance ahead, which is called look-ahead distance. Figure 17 represents the basic principle of Pure Pursuit. (X,Y) shows the current position of the vehicle. *L* gives the look-ahead distance, and goal point followed by the algorithm is denoted by $\left(X_{L},Y_{L}\right)$. The $R_{t}$ radius of the arc joining the current position to look-ahead distance. The following equation is satisfied based on two triangles.

(6) $$\begin{array}{cc} \; & X_{L}^{2}+Y_{L}^{2}=L^{2},\\ \; & o_{L}^{2}+Y_{L}^{2}=R_{t}^{2},\\ \; & x_{L}+o=R_{t}, \end{array}$$

The *o* in Equation (6) is put into Equation (7), resulting in:

(7) $$\;\begin{array}{cc} \; & {\left(R_{t}-x_{L}\right)}^{2}+Y_{L}^{2}=R_{t}^{2},\\ \; & R_{t}^{2}-2R_{t}x_{L}+X_{L}^{2}+Y_{L}^{2}=R_{t}^{2},\\ \; & R_{t}=\frac{L^{2}}{2x_{L}}. \end{array}$$

The $R_{t}$, arc radius defines the path followed by the autonomous vehicle.

Model predictive control (MPC) is another approach for designing controllers dealing with multivariable constrained control problems [66]. In MPC, an optimal control problem is repeatedly solved to minimize the objective function subject to constraints over the future horizon. The future response of the system is enumerated according to the model [67]. In the context of automotive control applications, MPC have been applied to braking and steering [68,69], traction control [70], lane-keeping [71]. MPC finds an optimal path by solving an optimization problem for each timestep and produces an actuator signal depending upon the finite horizon. The formulation of MPC optimization for lateral control is given below;

(8) $$\begin{array}{c} \min_{U}\sum_{i=0}^{H_{p}}\left[{\left(z_{i}-z_{ref,i}\right)}^{T}Q\left(z_{i}-z_{ref,i}\right)\right]+\sum_{i=0}^{H_{p}-1}\left[{\left(u_{i}-u_{i-1}\right)}^{T}\bar{R}\left(u_{i}-u_{i-1}\right)+u_{i}^{T}Ru_{i}\right] \end{array}$$

s.t

$$\begin{array}{l} z_{0}=z\left(t\right),u_{-1}=u\left(t-t_{s}\right),\\ z_{i+1}=f\left(z_{i},u_{i}\right),i=0,\ldots ,H_{p}-1\\ u_{min,i}⩽u_{i}⩽u_{max,i},\forall i{\dot{u}}_{min,i}⩽\frac{u_{i}-u_{i-1}}{t_{d}}⩽{\dot{u}}_{max,i},\forall i/0\\ {\dot{u}}_{min,i}⩽\frac{u_{i}-u_{i-1}}{t_{s}}⩽{\dot{u}}_{max,i},i=0\\ . \end{array}$$

The model is discretized, where $Q\in R^{4\times 4},\;R\in R^{2\times 2},\;\bar{R}\in R^{2\times 2},\;H_{p}$ is the horizon prediction. The diagonal matrix represent the cost weights $Q,\;R,\;\bar{R}$. The controller sampling time is $t_{s}$. The discretized system dynamics of the kinematics model is given by $f(z_{i},u_{i})$ where $u_{-1}$ is the input given to previous sampling step $u(t-t_{s})$. The constraints variables are given for lower and upper bound are given by $u_{min},\;{\bar{u}}_{max}$ and $u_{max},\;{\bar{u}}_{max}$ respectively. The reference trajectory is given by $z_{ref}$.

(9) $$\min_{U}\sum_{i=0}^{H_{p}}\left[{\left(z_{i}-z_{ref,i}\right)}^{T}Q\left(z_{i}-z_{ref,i}\right)\right]+\sum_{i=0}^{H_{p}-1}[R\left(\Delta {\left(F_{y_{f}}\left(k+i\right)\right)}^{2}+W\varepsilon_{v}\right],$$

where $\Delta F_{y_{f}}=\left[\Delta F_{y_{f}}\left(k\right),\Delta F_{y_{f}}\left(k+1\right),\ldots ,\Delta F_{y_{f}}\left(k+H_{p-1}\right)\right]$. $\varepsilon_{v}$ is the non-negative slack variable. Q,R and W are the weighting matrices.

#### 3.4.3. Experimental Results

**Figure 18:**
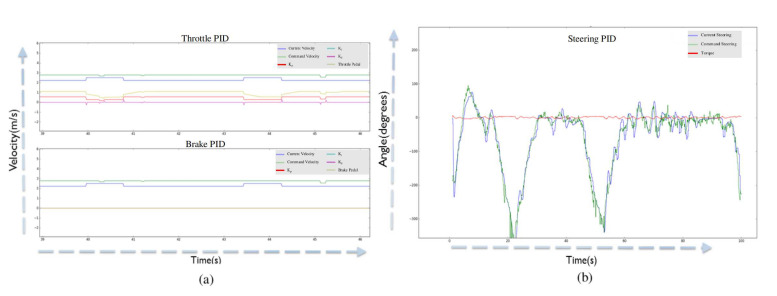
(**a**) The current velocity (from can-bus) and command velocity (from twist filter). It also shows the Throttle Pedal and PID parameters (Proportional, Integral and Derivative) (between 1 and −1). (**b**) The graph of current steering and target steering values along with the torque information [24] (Best viewed in color).

The lateral and longitudinal control is evaluated and experimented on the autonomous vehicle. The longitudinal control generates torque, throttle, and brake commands for the autonomous vehicle. The PID controller is employed, and its optimization parameters are given by Equation (5). These three parameters are proportional $\left(K_{p}\right)$, integral $\left(K_{i}\right)$, and derivative $\left(K_{d}\right)$, which are optimized for the robustness of the controller. We have used two techniques for tuning the parameters of PID, (i) Cohen-Coon method [72], and (ii) Genetic Algorithm (GA) [73,74]. In Cohen-Coon method [72], the proportional ($K_{p}$) parameter is tuned first by making the integral ($K_{i}$) and derivative ($K_{d}$) parameters zero. Once the $K_{p}$ is tuned, the other parameters are tuned accordingly. The PID tuned graphs are shown in Figure 18.

**Figure 19:**
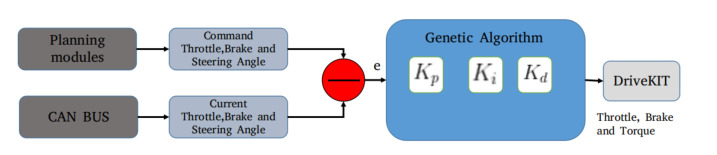
Framework for tuning the PID parameters using genetic algorithm is shown. The parameters are tuned for throttle, brake and steering respectively.

The tuning of parameters by Cohen-Coon method [72] is manual and requires human supervision in order to find optimal parameters. This problem of manual tuning of parameters is solved using the genetic algorithm as an optimization problem, constrained to minimize the error signal between current and command signal for throttle, brake and torque respectively [75]. Figure 19 illustrates the framework for optimizing the parameters using the genetic algorithm. Table 5 shows the quantitative comparison between genetic algorithm and Cohen-Coon method for obtaining the PID parameters. It shows that the parameters achieved using the genetic algorithm approaches to same parameters values as achieved by Cohen-Coon method but finding the optimal values using optimization without the need for manual tuning of PID parameters.

**Figure 20:**
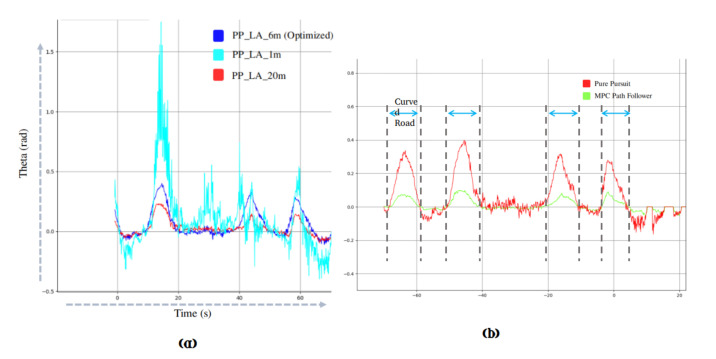
(**a**) illustrates the effect of look-ahead distance in pure pursuit. The look-ahead distance with different configuration is illustrated in the legend of the graph; for instance, the PP-LA-1m corresponds to 1m look-ahead distance in the pure pursuit. The look-ahead distance of 1m is more prone to noise, and produces more vibrations in the lateral control of the vehicle. Moreover, the look-ahead distance of 20 m, deviates the lateral control from the original track. The optimized look-ahead distance of 6m gives the optimal result with minimal error in contrast to former look-ahead distances. The steering angle for lateral control is shown by Theta (rad). (**b**) illustrates the graph of lateral error difference between MPC and pure pursuit [24] (Best viewed in color).

**Figure 21:**
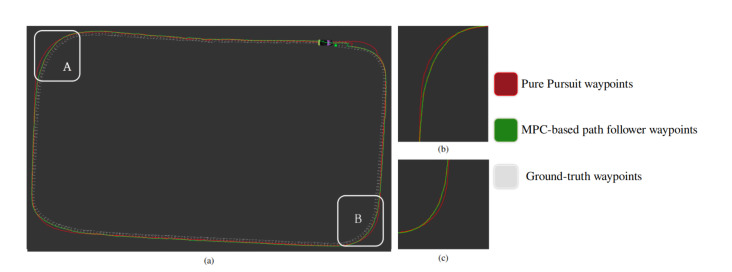
(**a**) illustrates the qualitative results between Pure Pursuit and MPC-based path follower. The difference between Pure Pursuit, and MPC-based path follower is shown in (**b**,**c**) respectively [24] (Best viewed in color).

For the lateral control of the autonomous vehicle, two path tracking algorithm are implemented and compared. The robustness of each controller is dependent upon optimal parameter tuning based on the vehicle model. For Pure Pursuit, the tuned parameters are given in the Table 6. The look-ahead distance is a primary parameter for Pure Pursuit, and we evaluated the performance by varying the look-ahead distance. Figure 20a shows the lateral control using Pure Pursuit. The small look-ahead distance leads to more disturbance and noise, whereas higher value causes the deviation from the specified path. In our previous work [24] the optimized parameters for MPC-based path follower are illustrated. The comparative analysis between Pure Pursuit and MPC path follower is performed by implementing the algorithm to the autonomous vehicle at a maximum speed of 30 km/h. Figure 21 shows the qualitative results. As visible from the Figure 21, in case of Pure Pursuit, the vehicle over-cut the defined path at corners, whereas MPC follows the ground truth more accurately. The quantitative analysis is performed by calculating the lateral error between Pure pursuit, and MPC-based path follower with ground truth as shown in Figure 20b. A significant difference is observed between two algorithms at curved roads; The MPC follows the path accurately, and does not overshoot as compared to Pure Pursuit, and thus ensures the safety of the autonomous vehicle when taking turns.

## 4. Experimental Validation: Autonomous Taxi Service

A large variety of usage scenarios and specifications exists for autonomous vehicle [76,77,78]. In this study for the experimental validation of the proposed autonomous vehicle, a cab booking service is implemented. The goal of this experimental validation is to test the whole autonomous driving stack extensively in an unconstrained environment with varying possible conditions on the course of the path like obstacle avoidance, emergency stopping and safely reaching to the destination as instructed by the customer.

**Figure 22:**
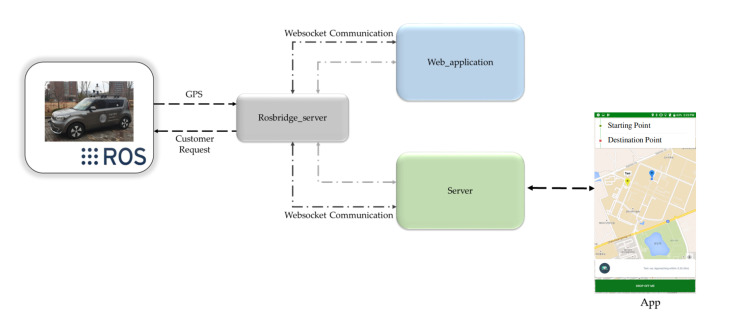
The framework of cab booking service (Best viewed in color).

**Figure 23:**
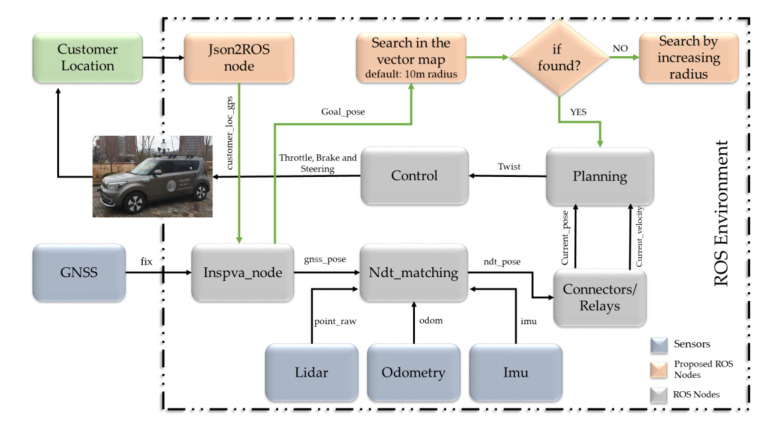
The detailed architecture of converting the autonomous vehicle to a cab service (Best viewed in color).

Figure 22 illustrates the overview of autonomous taxi service. An android app is developed that takes the request from the customers and send the location and destination of the customer to the autonomous vehicle through an intermediate booking server. For the communication between the autonomous vehicle and server, we adopted the ROSbridge that acts as a middleware between the ROS and web application. The GPS location of the autonomous vehicle is sent to the server through ROSbridge. The server is also responsible for getting the customer location through an android app and send it to an autonomous vehicle. A web application, communicate over WebSocket, on the autonomous vehicle side is created that functions as a status updater for autonomous vehicle and also for acknowledging the customer request. The architecture of the autonomous vehicle is modified to handle the incoming customer requests for autonomous taxi service. Figure 23 shows the modification perform in the stack of an autonomous vehicle in order to adapt it for cab service. The customer location is received by *Json2Ros* node. The *Json2Ros* node extract the GPS location of the customer and convert it to ROS format messaging system and publish it as cab GPS message. The *cabgps2tfpose* subscribed the cab GPS message and converted it to pose, which includes both position and orientation. This pose information is given to the stack of the autonomous car to describe customer location. The location of the customer is searched in the map, and if it is found, then the location is given to the planning module to find the path to the customer’s location from the current location. Besides, if the location is not perceived with the current configurations, then the search area is increased.

**Figure 24:**
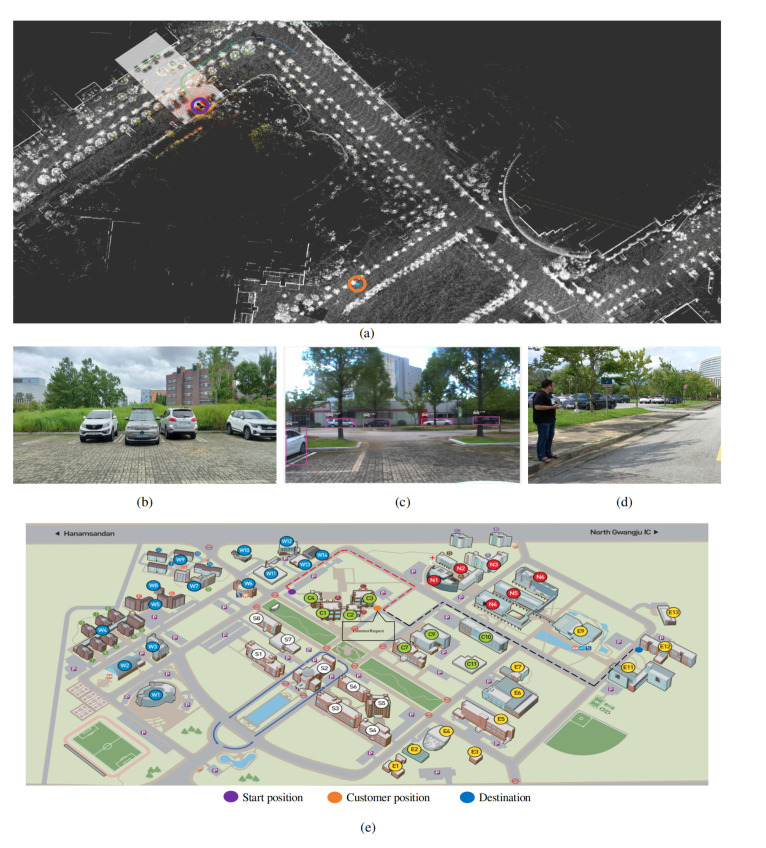
The visualization of autonomous taxi service as an application of our autonomous vehicle. (**a**) The 3D map shows the start and customer’s pick up position upon receiving the request. (**b**) Image view of start position. (**c**) Front-facing camera view from the start position. (**d**) illustrates that the customer is waiting for the autonomous taxi service. (**e**) The map of our institute shows the start, customer and destination position (Best viewed in color).

**Figure 25:**
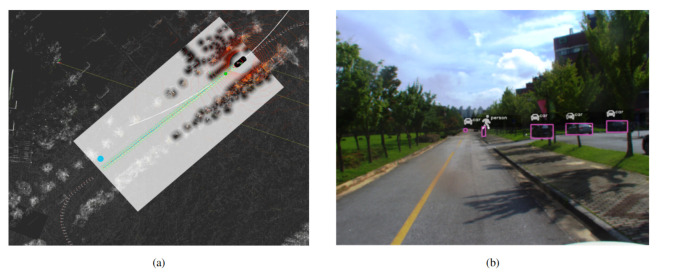

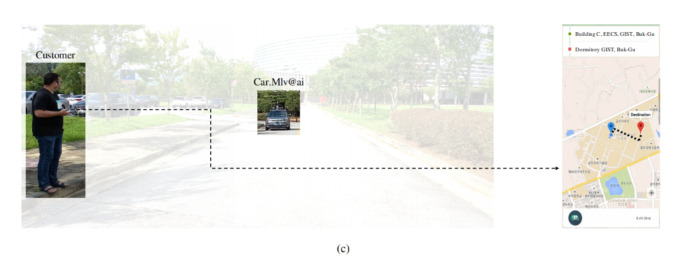
The visualization results of an autonomous vehicle approaching to pick up the customer. (**a**) The 3D map of the environment showing the ego vehicle and customer’s position. (**b**) The view of the customer from the autonomous vehicle front-facing camera. (**c**) illustrate the environment and also showing the app which customer is using to request for autonomous taxi service (Best viewed in color).

**Figure 26:**
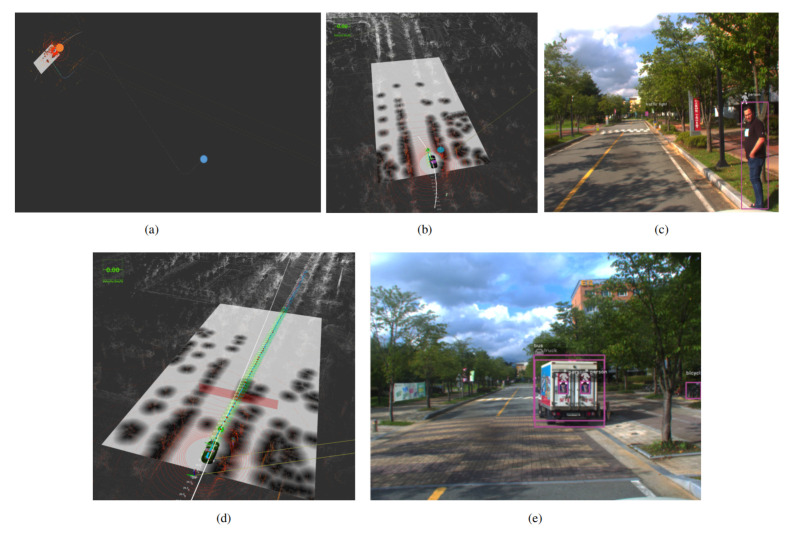
The route of the autonomous vehicle after picking the customer to the destination is illustrated in (**a**–**c**) along with object detection and autonomous vehicle stopping at the obstacle as shown in (**d**,**e**). In that particular case, obstacle avoidance is not performed because this is the one-side road and obstructing the yellow line is against the traffic rules (Best viewed in color).

Figure 24, Figure 25 and Figure 26 illustrate the results of autonomous taxi service (For the presentation constraints, the results are shown at the end of manuscript). The system is tested in our campus. The customer requests a cab service, which is then accepted by the autonomous vehicle if its available. The autonomous vehicle, after path planning it proceeds to the customer location by maintaining a maximum speed of 30 km/h. After picking up the customer, the path is planned for the customer destination, which is followed by the autonomous car, and the customer is dropped off safely to the destination.

## 5. Comparison

In order to construct a comparative analysis of Car.Mlv.ai in the context of hardware and software setups with other state-of-the-art autonomous vehicles, both research, and notable companies vehicles have been analyzed for comparison. For the hardware level comparison, the most notable state-of-the-art autonomous vehicle’s companies have been selected which have been tested in the real-world scenarios. As our autonomous vehicle is designed with the constrained resources, so the central aspect on which the comparison has done is the number of sensors used in these state-of-the-art autonomous vehicles. Table 7 shows a comparison on the hardware level. For instance, Waymo has used 5 Lidars, one medium-range at the top and four short-range Lidar for the front, left side, right side and rear along with 360 degrees camera at the top of the vehicle, and also GNSS, IMU as a supplement sensors in their hardware stack [14]. GM Chevy Bolt Cruise, on the other hand, have used five 32 channels Lidars with sixteen cameras for scanning 360 degrees long and short-range view around the vehicle. In addition, GM Chevy Bolt Cruise have also used 21 radars in their sensor’s stack for determination of precise speed [15]. Zoox has used 8 Lidars with 12 cameras along with radar, and GNSS+IMU system [79]. Similarly, Uber has used more than one Lidar and camera sensors in their hardware stack [12]. In contrast to these state-of-the-art autonomous vehicles, our autonomous vehicle’s hardware stack includes one 32 channels Lidar and 2 cameras along with navigational unit (GNSS+IMU) system reducing the cost as compared to other autonomous vehicles. The software stack comparison with aforementioned companies is hard to make because the information about their software stack is proprietary.

The Car.Mlv.ai is compared to other research vehicles in term of hardware and software level. Table 8 shows the hardware stack comparison of Car.Mlv.ai and other research vehicles. VaMP [6] developed by Thomanek and Dickmanns is equipped with four camera and INS system and is tested for adaptive cruise control and lane change only due to minimum sensor capabilities. The two winning teams from DARPA Urban Challenge and the Grand Cooperative Driving Challenge respectively are also included for the comparison. Boss [17] and Junior [7] have used Lidar and Radar fusion for detection and camera as a classification of obstacles in the urban environment because back then GPU-based acceleration and deep learning for perception modules have not been optimized to be tested on the autonomous vehicles because of computation constraints. Similarly, other research vehicles, for instance, RACE [8], Halmstad [9] and Bertha [18] have used advanced sensor suite. With reference to Apollo [10], GPS with Real-Time Kinematic (RTK) have been used for localization along with Lidar (64 channels) and cameras (front and side view) for the perception of the environment.

For the software stack comparison between Car.Mlv.ai and other research vehicles, we have focused on the application area of operation, operating system, middleware, functional safety, controller and licensing of the software of the autonomous vehicles. Since the middleware provides the basis for developing the software stack for an autonomous vehicle, nearly all the research vehicles have used some form of customized middleware layer for their autonomous vehicles. VaMP [6] and Bertha [11] have no information about their software architecture. At the time DARPA Urban Challenge, ROS was not released, though Boss [17] and Junior [7] have used publish/subscribe inter-process communication (IPC) system which is close to the structure of ROS. Bertha (2016) have used ROS as a middleware for their software stack. The first version of Apollo [10] have used an extended version of ROS with message protocol based on Google Protocol Buffers and decentralized node management but from version 3.5 Apollo is using Cyber RT a custom middleware instead of ROS. Halmstad [9] and RACE [8] have used Lightweight Communication and Marshalling and RACE RTK as their middleware for autonomous vehicle respectively. As compared to these research vehicles, we have used ROS for developing the software stack of our autonomous vehicle. In the context of the controller, each vehicle has used its designed controller for the vehicle. The comparison of other software aspects is shown in Table 9 between Car.Mlv.ai and other research vehicles.

## 6. Discussion

In designing the proposed autonomous vehicle, we have employed the ISO 26262 V framework to test and evaluate the proposed autonomous vehicle. In general, the V model represents the methodological process of developing the autonomous vehicle, followed by verification and validation. One of the crucial challenges faced by employing this model is a non-deterministic and statistical analysis of the autonomous vehicle. It is correct that some of the technologies used in the autonomous vehicles are statistical and tend to be non-deterministic, but it is difficult to reproduce, for instance, non-deterministic computation of planners. The handling of non-deterministic in testing is problematic for at least two reasons. First, it is difficult to reproduce a specific edge-case situation because the system might behave differently on the activation of that specific case upon receiving the world’s sequence inputs. The second case with non-determinism in testing is that it can be challenging to evaluate whether the test result is correct or not. The correctness criteria are only that if the autonomous vehicle ensures the safety of the environment or not, and if the safety is not violated and autonomous vehicle behavior is safe, it is considered a positive test. Also, multiple tests might be performed to ensure that vehicle always land in the test pass phase concerning the land in the test pass phase concerning the safety [80,81].

In the proposed autonomous vehicle, each modules are operated under some time constraints. The sensors data are operated at different frequencies. A detail of sensor’s operating frequencies have presented in the Table 10. Since the ROS follows the asynchronous protocol, the specific node is executed upon receiving all the essentials incoming messages. For the system latency, from sensor’s input receiving to output execution in an obstacle detection case, the whole task takes 1650 μs. In the current stack of proposed autonomous vehicle, the execution time of ROS nodes is less that 2000 μs driving at speed of 30 km/h. In addition, the distributed architecture over the Gigabit network, the use of Nvidia GPU for the object detection task and multi-tasking environment enable the execution of task in the proposed autonomous vehicle stack efficiently in real time.

The localization of the autonomous car is based on the 3D point cloud map. The NDT mapping is used to develop the 3D map. The data is collected using 32 channels Lidar. As the Lidar scans consist of 1.39 million points per second, around 132Gb of raw data is collected. The primary obstacle is to process this vast amount of data. In order to solve this problem, the data is divided into chunks covering an area of around 100 m. These individual chunks are used for making the separate maps, and they are combined together using the open-source software Cloud Compare (https://www.danielgm.net/cc/). The 3D map is moreover filtered using voxel filtering. The 3D map is built in Lidar reference frame; this 3D map is then transformed to world coordinate using the GPS data. The transformation is calculated by identifying key landmarks in point cloud map, and world coordinate from GPS data.

In order to get precise GPS and IMU data, we have installed a Novatel GNSS module in the autonomous vehicle. Novatel company provides software support for windows, and only a few open-source drivers are available for Linux operating system. Since our autonomous car is using ROS as middleware for the development of software stack; the GPS and IMU data are needed to be in the ROS format. In order to cater to this issue a Linux based driver was developed that runs the Real-time kinematic (RTK) client and convert the data to ROS format. The raspberry pi is attached directly to the GNSS module through USB, and it publishes the data to ROS master over Ethernet. Odometry data provides vital information for vehicle control. Here, the odometry data was obtained from CAN bus instead of IMU. The major disadvantage of using IMU is that it suffers from accumulated error. The velocity from IMU is continuously integrating over time, and small error are accumulated over time, and this creates the problem of drift, whereas velocity from CAN bus is accurate. Drivekit does not provide odometry data. We have used CAN-BUS shield to read messages from CAN bus. We decoded CAN message corresponding to odometry data empirically because the ID’s of the CAN bus is proprietary information and the automotive company does not provide this information.

The detection module consists of fusion of exteroceptive sensor data by performing object detection using and Lidar, and camera. All the processing is needed to be performed in real-time. The Lidar has a frame rate of 15 Hz, which set a maximum limit for processing the raw data. The detection of the object in images is done by using YOLOv3 which gives processing frame rate of 15 Hz. We have opted to use PointPillars for detecting the objects in Lidar. An Intel core i7 processor with TITANX GPU having 47GB memory, is used for detection and fusion module. The fusion of data is computationally inexpensive as only detected object bounding boxes are transformed into a common frame. The data fusion also provides the reliability of data by compensating the weakness of one sensor with the other. The camera provides spatial information, and the Lidar receives depth information, so the fusion of these two gives both the spatial and depth information about the surrounding environment. Another major challenge is to compensate for the computation power required by the fusion. In our scenario, we split the process into two systems. The Lidar-based detection, and fusion is performed on one system that runs the ROS-master. The other system is laptop which also works as a front-end for the user, and also performs the object detection in images, and publishes the detected object message to the ROS-master. In this distributed system, the latency, and computation issues are resolved.

The parameter tuning for planning module includes stopping distance, stopping acceleration limit, and obstacle avoidance velocity limit. All the aforementioned parameters are tuned manually by testing in real-time, and are optimized for our autonomous vehicle. A customized PID controller is designed for our autonomous vehicle. It consists of a PID controller for each torque, brake, and throttle. The PID parameters are sensitive, and they were tuned for specific maximum speed using the Cohen-Coon method and genetic algorithm. There are three ranges of speed, 0–10 km/h, 10–30 km/h and 30–60 km/h. The obtained parameters were extensively tested.

## 7. Conclusions

This study developed an autonomous vehicle with a minimal sensor suite and designed a distributed architecture for the proposed autonomous vehicle. The autonomous vehicle architecture constitutes the sensors layer, perception layer, planning layer, and control layer. The integration of exteroceptive and proprioceptive sensors, data fusion of exteroceptive sensor for the perception of the environment, and state-machine design for optimal trajectory generation by complying the traffic rules and a customized controller for longitudinal and lateral control are experimentally evaluated in an autonomous taxi service. The autonomous vehicle is tested in several scenarios and has successfully performed stopping at the obstacle in the two-way single-lane road, obstacle avoidance, complying with traffic rules, collision avoidance, and keeping in the lane. The experimental analysis of proposed modules in each layer is evaluated in a real-time operating system. Also, the system’s latency corresponding to each ROS topic is evaluated in the proposed work.

Future work includes the integration of ultrasonic sensors to detect small objects near the vehicle and the autonomous parking. Furthermore, audio-visual information inclusion to detect an emergency vehicle and ego-lane change is part of future work. In addition, the voice activation of receiving the command from the passenger for the destination is included in the future work.

## Figures and Tables

**Table 1:** Normal Distribution Transform (NDT) Matching Experimental Parameters.

Parameters	Values
Error Threshold	1
Transformation Epsilon	0.1
Max Iteration	50

**Table 2:** Quantitative comparison between GNSS pose and NDT pose. RMSE is computed in x, y, and z direction respectively. The overall RMSE is also computed between the two poses.

Error between GNSS Pose and NDT Pose	RMSE
x-direction	0.2903
y-direction	0.0296
z-direction	0.0361
Overall	1.7346

**Table 3:** Ground Plane Removal Filter Experimental Parameters.

Parameters	Values
Sensor height	1.8
Distance Threshold	1.58
Angle Threshold	0.08
Size Threshold	20

**Table 4:** Experimental Parameters for Planning Layer.

Parameters	Values
Stop distance for obstacle Threshold	10
Velocity replanning distance Threshold	10
Detection range	12
Threshold for objects	20
Deceleration for obstacle	1.3
Curve Angle	0.65

**Table 5:** Quantitative comparison of Cohen-Coon method and Genetic algorithm for tuning the PID parameters. Cohen-Coon method is manual tuning as compared to Genetic algorithm in which the tuning of PID parameters are obtained by defining the objective function and solve as an optimization problem [24].

Controller Parameters	Throttle		Steering	
	Cohen-Coon Method	Genetic Algorithm	Cohen-Coon Method	Genetic Algorithm
$K_{p}$	0.085	0.003	0.0009	0.0005
$K_{i}$	0.0045	0.0001	0.0001	0.0002
$K_{d}$	0.01	0.09	0.0005	0.0008

**Table 6:** Pure pursuit tuned parameters for 30 km/h speed.

Pure Pursuit Parameters	Values
Look-ahead ratio	2
Minimum Look-ahead Distance	6

**Table 7:** Hardware level stack comparison between state-of-the-art and our autonomous vehicle in term sensor utilization.

Hardware Level Stack Comparison					
**S.No**	**Autonomous Vehicles**	**Lidar Units**	**Camera Units**	**Supplement Sensors (GNSS,IMU, Radars, Sonars)**	
1.	Waymo [14]	5	1	GNSS+IMU	
2.	GM Chevy Bolt Cruise [15]	5	16	Radars	
3.	ZOOX [79]	8	12	Radar+GNSS+IMU	
4.	Uber(ATG) [12]	7	20	Radars, GNSS+IMU	
6.	Ours (Car.Mlv.ai)	1	2	GNSS+IMU	

**Table 8:** Hardware comparison of the relevant research-based autonomous vehicle is shown. ● indicates an existing component, ❍ shows the nonexistence of specific component and ? explains that the required information is not public. For reference, DARPA challenges and Apollo are considered for comparison.

	1994	2007		2013	2015	2016		2018	2019
Sensors	VaMP [6]	Junior [7]	Boss [17]	Bertha [11]	Race [8]	Halmstad [9]	Bertha [18]	Apollo [10]	Ours (Car.Mlv.ai)
Camera	● front/rear	❍	● front	● stereo	● front	❍	● stereo/360 deg	● front/side	● front
Lidar	❍	● 64 channels	●	❍	● 4 channels	❍	● 4 channels	● 64 channels	● 32 channels
Radar	❍	●	●	●	●	● series	●	●	●
GPS	❍	●	●	●	●	● rtk	●	● rtk	● rtk
INS	●	●	●	?	●	●	●	●	●
PC	●	●	●	?	●	●	●	●	●
GPU	❍	❍	❍	?	❍	❍	●	●	●

**Table 9:** Software comparison of relevant research based autonomous vehicles. ? illustrates that the required information is not public.

	1994	2007		2013	2015	2016		2018	2019
	**VaMP [6]**	**Junior [7]**	**Boss [17]**	**Bertha [11]**	**Race [8]**	**Halmstad [9]**	**Bertha [18]**	**Apollo [10]**	**Ours (Car.Mlv.ai)**
Application	German Highway	DARPA Urban Challenge		German Rural	Parking	Cooperative Driving Challenge		Various	Various but focus on Autonomous cab services
Middleware	?	Publish/Subscribe IPC	Publish/Subscribe IPC	?	RACE RTE	LCM	ROS	Cyber RT	ROS
Operating System	?	Linux	?	?	Pike OS	Linux	Linux	Linux	Linux
Functional Safety	None	Watchdog module	Error Recovery	?	Supporting ASIL D	Trust System	?	System Health Monitor	Supporting ASIL D
Controller	?	PC	?	?	RACE DDC	Micro Autobox	realtime onboard comp.	PC	DriveKit and on board controller
Licensing	Proprietary	Partly open	Proprietary	Proprietary	Proprietary	Proprietary	Proprietary	Open	Open

**Table 10:** The details of sensors operating frequency.

Sensors	Frequency (Hz)
Lidar	15
Camera	25
GPS	20
Can info	200
IMU	20
odomerty	200

## Abbreviations

The following abbreviations are used in this manuscript:

SOTIF	Safety of the intended functionality
DARPA	The Defense Advanced Research Projects Agency
SAE	The Society of Automobile Engineers
SLAM	Simultaneous localization and mapping
NDT	Normal Distribution Transform
RTK	Real-time kinematics
IMU	Inertial Measurement Unit
GNSS	Global Navigation and Satellite System
CAN	Controller Area network
GPS	Global Positioning System
ROS	Robot Operating System
SPI	Serial Peripheral Interface
